# An Asymmetrically Balanced Organization of Kinases versus Phosphatases across Eukaryotes Determines Their Distinct Impacts

**DOI:** 10.1371/journal.pcbi.1005221

**Published:** 2017-01-30

**Authors:** Ilan Smoly, Netta Shemesh, Michal Ziv-Ukelson, Anat Ben-Zvi, Esti Yeger-Lotem

**Affiliations:** 1 Department of Computer Science, Ben-Gurion University of the Negev, Beer-Sheva, Israel; 2 National Institute for Biotechnology in the Negev, Ben-Gurion University of the Negev, Beer-Sheva, Israel; 3 Department of Life Sciences, Ben-Gurion University of the Negev, Beer-Sheva, Israel; 4 Department of Clinical Biochemistry and Pharmacology, Ben-Gurion University of the Negev, Beer-Sheva, Israel; Massey University, NEW ZEALAND

## Abstract

Protein phosphorylation underlies cellular response pathways across eukaryotes and is governed by the opposing actions of phosphorylating kinases and de-phosphorylating phosphatases. While kinases and phosphatases have been extensively studied, their organization and the mechanisms by which they balance each other are not well understood. To address these questions we performed quantitative analyses of large-scale 'omics' datasets from yeast, fly, plant, mouse and human. We uncovered an asymmetric balance of a previously-hidden scale: Each organism contained many different kinase genes, and these were balanced by a small set of highly abundant phosphatase proteins. Kinases were much more responsive to perturbations at the gene and protein levels. In addition, kinases had diverse scales of phenotypic impact when manipulated. Phosphatases, in contrast, were stable, highly robust and flatly organized, with rather uniform impact downstream. We validated aspects of this organization experimentally in nematode, and supported additional aspects by theoretic analysis of the dynamics of protein phosphorylation. Our analyses explain the empirical bias in the protein phosphorylation field toward characterization and therapeutic targeting of kinases at the expense of phosphatases. We show quantitatively and broadly that this is not only a historical bias, but stems from wide-ranging differences in their organization and impact. The asymmetric balance between these opposing regulators of protein phosphorylation is also common to opposing regulators of two other post-translational modification systems, suggesting its fundamental value.

## Author Summary

Protein phosphorylation is a reversible modification that underlies cellular responses to stimuli across organisms. Historically, the study of protein phosphorylation concentrated on the role of kinases, which introduce the phosphate, at the expense of phosphatases, which remove it. Many kinases have been associated with specific phenotypes and considered attractive drug targets, while phosphatases remained far less characterized. It has been unclear whether this discrepancy is due to historical biases or reflects real systemic differences between these enzymes. By analyzing large-scale ‘omics’ datasets across genes, transcripts, proteins, interactions, and organisms, we uncovered an asymmetric architecture of kinases versus phosphatases that balances between them, determines their distinct impact patterns, and affects their therapeutic potential. This architecture is conserved from yeast to human and is partially shared by two other protein modification systems, suggesting it is a general feature of these systems.

## Introduction

Protein phosphorylation is a common post-translational modification, in which a phosphate group is covalently attached to amino acid residues within a protein by the function of a kinase. The phosphorylated protein may acquire a different reactivity, interaction specificity or cellular localization, which allow it to carry functions that the unmodified protein could not [1]. Protein phosphorylation is reversible, and upon removal of the phosphate group by the function of a phosphatase, the de-phosphorylated protein regains its previous functionality [2–4]. Reversible protein phosphorylation underlies signal transduction and controls main cellular processes across eukaryotes, such as cell-cycle, metabolism, transcription and translation [5]. In humans, about 30% of the proteins undergo phosphorylation, many of which in a reversible manner, and abnormal phosphorylation has been associated with complex diseases, cancers, and pathogen infection [3]. Protein phosphorylation is therefore under strict regulation, and is governed by the balanced actions of kinases and phosphatases.

The critical role of protein phosphorylation led to extensive studies of kinases and phosphatases, including large-scale 'omics screens that were carried predominantly in budding yeast (e.g., [6–9]). By using mass spectrometry to analyze kinase and phosphatase interactions, Breitkreutz et al [7] showed that an extensive backbone of kinase-kinase interactions cross-connects the yeast proteome. The profiling of strains carrying inactivated kinases or phosphatases using epistatic mini-arrays [6], mRNA profiling [8], and phospho-proteomic screens [9] revealed numerous functional overlaps and regulatory relationships among kinases and phosphatases. However, these and other meta-analyses (e.g., [1, 10]) often treated kinases and phosphatases as one group, although their internal organization might differ.

Here, we harnessed 'omics' datasets that were gathered from budding yeast, fly, plant, mouse and human, to gain insight into the functional organization of kinases and phosphatases. In particular, we asked how their organization supports the specific, transient and robust response to signals and perturbations. The field of cell signaling has been discussing these issues for a long time [11], and answers have been given mostly for a small set of well-studied kinases and phosphatases [11–12]. Our goal here was to contribute to this discussion by adding a quantitative, wide-ranging perspective across different proteins and different organisms, which has been partial so far. We were able to obtain this quantitative view by meta-analyses of diverse sets of large-scale data. We found that kinases and phosphatases are organized in distinct ways that are conserved across eukaryotes. Firstly, they maintain a quantitative balance, where kinases have many more genes while the few phosphatase proteins are more abundant. Secondly, they have a different responsiveness behavior, to which we provide a dynamics-based theoretic insight. Thirdly, they have a different impact behavior downstream, which we demonstrate experimentally by using the development of the vulva in *Caenorhabditis elegans* as readout. Lastly, we show that some of these features are also shared by the opposing regulators of histone acetylation and of protein ubiquitination in budding yeast, suggesting that these features are inherent to reversible post-translational systems.

## Results

### Intriguing asymmetries between kinases and phosphatases at the gene and protein levels

We analyzed kinases and phosphatases from the model organisms *Saccharomyces cerevisiae*, *Arabidopsis thaliana*, *Drosophila melanogaster* and *Mus musculus*, and from *Homo sapiens*. These organisms were selected because their kinases and phosphatases were screened in an unbiased manner and because they span different evolutionary routes. Notably, our analyses henceforth focus on the catalytic subunits of kinases and phosphatases (see Materials and Methods), and do not refer to regulatory or inhibitory subunits of these enzymes, for which less data are available.

**Fig 1:**
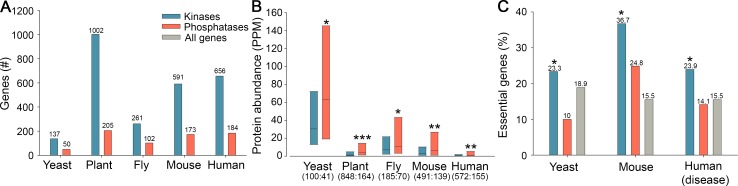
Differences in gene numbers, protein abundance and essentiality between kinases and phosphatases are conserved across eukaryotic lineages. A. Kinase-coding genes are more abundant than phosphatase-coding genes in five eukaryotic genomes. B. Phosphatase proteins are significantly more abundant than kinase proteins in five eukaryotic proteomes. Box plots show the values at the first, second and third quartiles. In parenthesis per organism are the numbers of kinases and phosphatases for which data were available. Median values for kinases and phosphatases, and the respective Mann-Whitney p-values, were as follows: Yeast 30.4, 63, p = 0.008; plant 1.3, 4, p<10^−10^, fly 7.5, 10.6, p = 0.03; mouse 2.8, 6.3, p = 9*10^−4^; human 0.33, 0.65, p = 5*10^−4^. C. The fraction of phosphatases that are essential for survival in yeast and mouse, or are associated with genetic disease in human, is significantly smaller than the fraction of kinases (yeast p = 0.03, mouse p = 0.0022, human p = 0.0023; Fisher exact test). Yeast = *Saccharomyces cerevisiae*; Plant = *Arabidopsis thaliana*; Fly = *Drosophila melanogaster*; Mouse = *Mus musculus*; Human = *Homo sapiens*. *** indicates p<10^−6^; ** indicates p<10^−3^; * indicates p<0.05. Numbers above bars indicate Y-axis values.

We first compared between the numbers of genes coding for kinases versus phosphatases in these eukaryotes. We found large discrepancies: The genome of budding yeast contains 137 kinases and only 50 phosphatases (a ratio of ~2.7:1), the human genome contains 656 kinases and only 184 phosphatases (a ratio of ~3.5:1), and similar preference toward kinases is present in the other genomes that we examined (Fig 1A).

How can the small pool of phosphatases revert the actions of the dominating sets of kinases? To answer this question we turned to protein levels (Fig 1B). Large-scale measurements of protein abundance [13] revealed that phosphatase proteins were roughly twice more abundant than kinases, a difference that was statistically significant across phyla (p≤8.3*10^−3^ in yeast, plant, mouse and human, and p = 0.036, in fly, Mann-Whitney). Thus, phosphatases seem to balance their reduced gene numbers by high protein abundance, consistently across diverse eukaryotes. We also tested tyrosine kinases and phosphatases, which constitute small subsets relatively to serine-threonine kinases and phosphatases and are known to have distinct characteristics [14], and obtained similar results (S1 Fig).

The relative scarcity in phosphatase genes may suggest that individual phosphatases are critical for viability and health. To test this we compared between the fractions of kinases and phosphatases that were shown to be essential for survival (Fig 1C). We observed the opposite trend: In budding yeast 23% of the kinases were essential, a fraction that is slightly higher than that of protein-coding genes (18.9%). Yet, only 10% of the phosphatases were essential (Fisher exact test, p = 0.03). For fly and plant such large-scale catalogs of essential genes were not available. In mouse 36.7% of the kinases were associated with a lethal phenotype [15], relative to 24.8% of the phosphatases (Fisher exact test, p = 2.18*10^−3^). For humans, we analyzed the set of over 3,100 genes that were genetically associated with a human disease [16]. Again, kinases were much more critical: 24% were associated with disease, relative to 14.1% of the phosphatases (Fisher exact test, p = 2.3*10^−3^). Thus, the numerous kinases have a strong effect on viability and health, while the fewer phosphatases appear to be much more functionally redundant.

### Kinases are more responsive than phosphatases at the gene and protein levels

Kinases and phosphatases are key components in signaling and cellular response pathways across eukaryotes. Therefore, we analyzed their ability to respond to perturbations. We focused on their tendency to alter their expression levels, to interact with other proteins, and to undergo phosphorylation.

**Fig 2:**
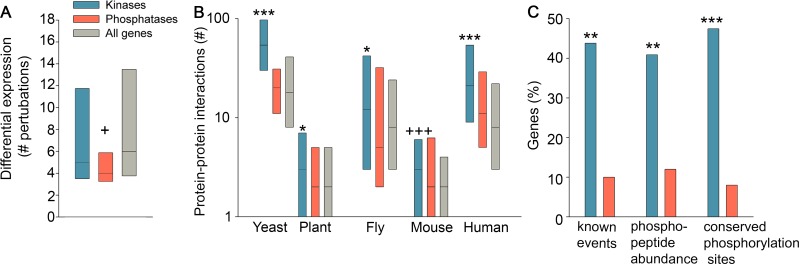
Kinases genes and proteins are more responsive than phosphatases. A. Yeast phosphatases are differentially expressed in fewer perturbations relative to all genes in yeast (p = 0.032, Mann-Whitney test). The data refer to the 45 kinases, 15 phosphatases, and 2979 protein-coding genes that were differentially expressed in at least one perturbation. B. Kinases are significantly more involved in protein-protein interactions (PPIs) relative to phosphatases (*) or to all proteins (+). The numbers of kinases, phosphatases, and protein-coding genes for which PPI data were available, and the respective Mann-Whitney p-value per organism, were as follows: Yeast 135, 49, 4,925, p<10^−10^; plant 377, 89, 6,430, p = 0.008; fly 213, 85, 9,539, p = 0.032; mouse 311, 52, 5,527, p = 2*10^−9^; human 633, 177, 16,387, p = 9*10^−7^. C. The capacity of yeast kinases to undergo phosphorylation is significantly higher than the capacity of phosphatases. This is observed in data of manually-curated phosphorylation / de-phosphorylation interactions (denoted known events), p = 5.7*10^−6^; in phospho-peptide abundance measurements, p = 9.7*10^−5^; and in a dataset of conserved phosphorylation sites within protein sequences, p = 1.4*10^−7^ (Fisher exact test). Box plots show the values at the first, second and third quartiles. ***/+++ indicate p<10^−6^; ** indicate p<10^−3^; */+ indicates p<0.05.

To test for changes in gene expression levels, we used transcriptional profiles of over 1,400 perturbation experiments in budding yeast [17]. About 34% of the yeast kinases and phosphatases were differentially expressed in at least one perturbation, a fraction that was significantly smaller than all genes (44%, p = 1.9*10^−3^, Fisher exact test, S2A Fig). Among those differentially expressed genes, phosphatases changed in significantly fewer perturbation experiments relative to all genes (p = 0.032, Mann-Whitney, Fig 2A), and in fewer perturbation experiments relative to kinases (p = 0.073). Notably, there was no correlation between the number of perturbations in which a gene was differentially expressed and its protein expression levels, and thus the reduced responsiveness of phosphatases could not be attributed simply to their higher abundance (see Materials and Methods). We next tested for differences in the protein stability of kinases and phosphatases by examining protein half-life measurements [18]. The protein half-life of kinases was significantly short (p = 2.7*10^−3^, Mann-Whitney test, S2B Fig), while that of phosphatases was similar to all genes, supporting the more dynamic nature of kinases.

One of the main ways by which kinases and phosphatases respond to perturbations is by interacting with other proteins. To assess their capacity for protein-protein interactions (PPIs), we gathered data of experimentally detected PPIs within each organism (see Materials and Methods). In all organisms, kinases were significantly more involved in PPIs than other proteins (Fig 2B), as was previously shown for yeast alone [7]. In yeast and human, which had the largest PPI coverage, kinases were also more involved in PPIs relative to phosphatases, supporting their increased responsiveness.

Lastly, we tested the capacity of kinases and phosphatases to undergo protein phosphorylation. For this we used three types of data: data of manually curated phosphorylation or de-phosphorylation interactions [6], measurements of changes in phospho-peptide abundance upon perturbation [9], and a dataset of conserved phosphorylation sites between yeast and human [19] (Fig 2C). In all datasets, over 40% of the kinases showed capacity for phosphorylation, relative to at most 12% of the phosphatases (p≤9.8*10^−5^, Mann-Whitney test). The observation that phosphatases are less phosphorylated than kinases is not necessarily expected from their different enzymatic activities [20]. In fact, some well-studied phosphatases, such as the family of CDC25 dual specificity phosphatases that regulates cell cycle [21], and the tyrosine phosphatase PTP-1B that regulates insulin and leptin signaling [22], have been shown to be heavily regulated by phosphorylation. Yet, our analysis shows that, as a group, phosphatases have a lower capacity for undergoing phosphorylation. In summary, the different analyses we carried suggest that kinases have a significantly higher tendency and capacity to respond to signals at the gene and protein levels, while phosphatases seem relatively static.

### A layered architecture of kinases and a flat organization of phosphatases

**Fig 3:**
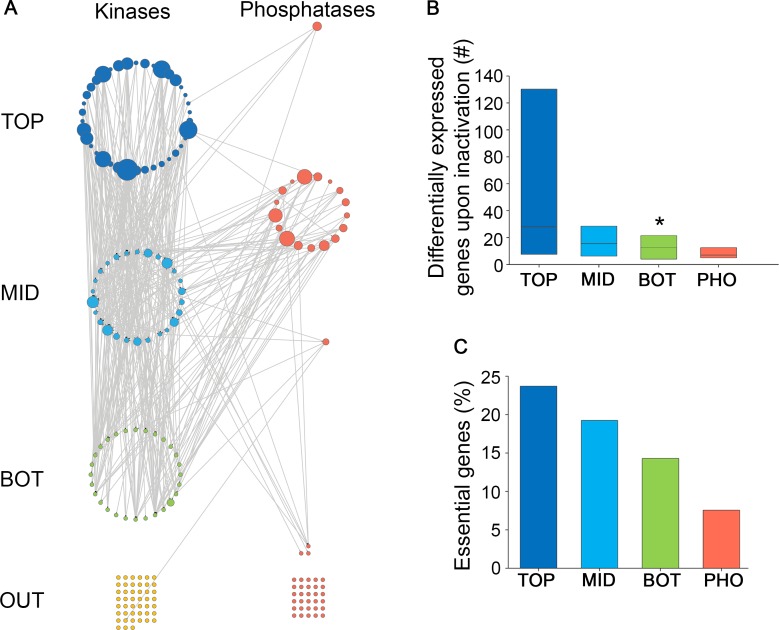
A hierarchical organization of yeast kinases by their impact. A. A layered architecture of kinases by their impact on the phosphorylation of each other. Impact edges point from the inactivated kinase (or phosphatase) down to its differentially-phosphorylated targets. Kinases and phosphatases with no impact relationship to other kinases / phosphatases appear as grids at the bottom. Kinases in the top, middle and bottom layers appear as blue, light-blue or green nodes, respectively; phosphatases appear in red. Node sizes reflect the number of target proteins. B. The impact of kinases and phosphatases on gene expression. Impact of an individual kinase (or phosphatase) was defined as the number of genes that were differentially expressed upon kinase (or phosphatase) inactivation. The impact of kinases from each layer decreased upon moving down the hierarchy, with bottom-layer kinases affecting the expression of significantly smaller sets of genes (p = 0.037, Mann-Whitney test). Data were available for 14 top-layer, 10 middle-layer, and 10 bottom-layer kinases, and for 18 phosphatases. Box plots show the values at the first, second and third quartiles. C. The phenotypic impact of kinases and phosphatases, as measured by the percentage of essential genes in each subset. The phenotypic impact of kinases decreased upon moving down the hierarchy. TOP = top layer, MID = middle layer, BOT = bottom layer, OUT = outgroup, PHO = phosphatases. * indicates p<0.05.

The large numbers of distinct kinase genes per organism led us to hypothesize that kinases may be organized in a hierarchical manner. Hierarchical analysis was used previously to organize transcription factors [23–26], and a unified set of kinases and phosphatases, based on their regulatory relationships [10]. Here, we defined relationships between kinases by their impact on the phosphorylation of each other, which were measured in a series of phospho-proteomic screens of budding yeast [9]. There, a kinase was considered to impact a target protein when the inactivation of the kinase resulted in the differential phosphorylation of the target protein. We modeled these impact relationships between kinases as a network, where nodes represent kinases and directed edges point from the impacting kinase to its target kinase. We then created a hierarchy of kinases by their impact relationships (Fig 3A). The top layer contained 38 kinases that impact other kinases but are not targeted by any kinase. The middle layer contained 26 kinases that are both targeted by kinases and also impact other kinases. The bottom layer contained 28 kinases that are targeted by other kinases and do not impact any kinase. 45 kinases without any kinase impact relationships were termed 'outgroup' and were omitted from further analyses.

To validate the resulting kinase hierarchy we used additional phosphorylation-related datasets. These included kinase impacts on the entire proteome [9], manually-curated phosphorylation interactions [6], the presence of experimentally verified phosphorylation sites within kinases [27], as well as the presence of conserved, and thus more likely functional, phosphorylation sites [19]. As shown in S3 Fig, in each dataset the different layers had distinct behaviors that were consistent with the impact-based organization of kinases. For example, top-layer kinases had significantly few conserved phosphorylation sites, whereas bottom-layer kinases had significantly more such sites, supporting their different tendencies to undergo phosphorylation (S3D Fig).

We next analyzed the impact of kinases on downstream targets using other types of data. For that, we analyzed the impact of kinases on gene expression [17]. Inactivation of kinases from the top layer affected the expression of the largest sets of genes, and this effect decreased upon moving down the hierarchy (Fig 3B). We also tested the impact of kinases on survival. The top layer had the largest fraction of kinases that were essential for survival, while the bottom layer had the lowest fraction (Fig 3C). The distinct features of each layer were preserved upon refining the hierarchy (see Materials and Methods, and S4 Fig and S5 Fig). Thus, while phospho-proteomic data may have caveats, comparisons to several other types of data show that upper layers indeed have broader impacts downstream.

Unlike kinases, phosphatases did not show a layered architecture. Phosphatases were involved in few intra-phosphatase impact relationships, and, in accordance with the kinase hierarchy, mainly affected kinases in the middle and bottom layers (Fig 3A). Phosphatases had limited impact on gene expression (Fig 3B) and on survival (Fig 3C). Thus kinases and phosphatases differ in their organization and impact behavior in budding yeast.

We turned to the nematode *C*. *elegans* to experimentally compare the impact of kinases and phosphatases on a specific phenotype, using the well characterized vulva differentiation system. The development of the vulva in *C*. *elegans* is regulated by epidermal growth factor (EGF) activation of RAS, WNT/beta-catenin and Notch signaling pathways [28]. Disrupting these signaling cascades can lead to decreased vulva induction causing a Vulvaless (Vul) phenotype, or activation of vulva induction, resulting in Multivulva (Muv) phenotype. The *let-60(ga89)* (*C*. *elegans* RAS) and *bar-1(ga80)* (*C*. *elegans* beta-catenin) alleles show low penetrance of Vul or Muv phenotypes depending on cultivation condition [29]. Thus, they provide sensitize backgrounds to explore and compare between the impact of various kinases and phosphatases that were previously associated with disruption of vulva development.

**Fig 4:**
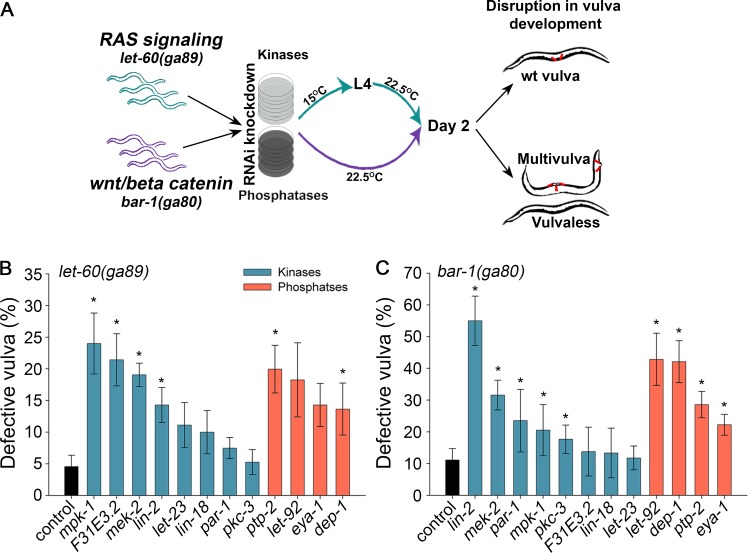
RNAi knockdown of *C*. *elegans* kinases results in variable effects on vulva development, while knockdown of *C*. *elegans* phosphatases results in more uniform effects. A. Illustration of the experimental pipeline. Age-synchronized animals were grown on kinase/phosphatase specific RNAi-expressing bacteria at the indicated conditions. The percentage of animals showing a disrupted vulva development (Multivulva / Vulvaless) was scored at day 2 of adulthood. B. For each RNAi-treatment of a kinase (or phosphatase) in animals with perturbed RAS signaling, the percentage of animals with disrupted vulva phenotype was scored. Shown are the medians (± SEM) of at least 3 independent experiments. Four kinase treatments and two phosphatase treatments showed a significantly higher tendency for disrupted phenotype relative to control (p-values: *mpk-1* p = 0.012, F31E3.2 p = 0.024, *mek-2* p = 0.024, *lin-2* p = 0.02, *ptp-2* p = 4*10^−3^, *dep-1* p = 0.046; Mann-Whitney test). C. Same as in B, for animals with perturbed wnt/beta-catenin signaling. Five kinase treatments and four phosphatase treatments showed a significantly higher tendency for disrupted phenotype relative to control (p-values: *lin-2* p = 2.2*10^−3^, mek-2 p = 0.013, *par-1* p = 0.018, *mpk-1* p = 0.043, *pkc-3* p = 8.6*10^−3^, *let-92* p = 4.6*10^−3^, *dep-1* p = 0.017, *ptp-2* p = 0.027, *eya-1* p = 0.023; Mann-Whitney test).

Similarly to the other eukaryotes that we examined (Fig 1A), *C*. *elegans* had many more genes coding for kinases than phosphatases (455 kinases relative to 177 phosphatases, a ratio of ~2.6:1). To assemble an extensive list of kinases and phosphatases that were previously associated with vulva phenotypes, we retrieved from WormBase all the kinases and phosphatases that were annotated with Vul or Muv phenotypes in any genetic background [30]. This resulted in eight kinases and four phosphatases. In wild type background, the knockdown by RNAi of any of these kinases and phosphatases did not result in vulva phenotypes, stressing the robustness of vulva development [30]. We then repeated this experiment in a RAS or beta-catenin mutant background (Fig 4A). In the RAS mutant, background treatment with empty vector control showed defective vulva (Vul or Muv phenotypes) in 4.6±1.8% of the *let-60(ga89)* animals. RNAi knockdown of individual kinases resulted in a wide differential scale of phenotypic impact, ranging from strong to no significant impact (Fig 4B). Of note, three of the four significant responder kinases, *mek-2*, *mpk-*1 and *lin-2*, are known members of the RAS/LET-60 vulva-signaling cascade [30], validating our analysis. In contrast, the four phosphatases had a medium impact with no significant difference between any two phosphatases examined (p>0.11, Mann-Whitney test). In the beta-catenin mutant background, treatment with empty vector control showed defective vulva in 11.1±3.6% of the *bar-1(ga80)* animals (Fig 4C). RNAi knockdown of individual kinases again resulted in wide scale of phenotypic impact, but the significant responders varied from the RAS mutant background. Similarly to the RAS mutant background, all four phosphatases had a medium impact. Thus, these data suggest that distinct kinases impact the output of a specific signaling pathway. In contrast, any given phosphatase resulted in a comparable phenotype, suggesting that in a sensitized background, phosphatases are interchangeable while kinases less so.

The observation that phosphatases had a noticeable phenotype in *C*. *elegans* may seem surprising given their lower essentiality rate (Fig 1C). However, essentiality was measured in a non-sensitized background, while in the *C*. *elegans* screen we used sensitized backgrounds. We, therefore, turned back to yeast to examine if sensitized background can impact phosphatases requirement. A negative genetic interaction (NGI) is called between two genes, *g1* and *g2*, when the strain carrying both deletions shows reduced growth relative to expectation based on the growth of a strain carrying a *g1* deletion and a strain carrying a *g2* deletion. Using data from a screen for NGIs involving kinases or phosphatases [6], we found that yeast phosphatases were slightly more involved in NGIs than kinases: 74% of the yeast phosphatases had at least one NGI, compared to 67% of the kinases. Additionally, phosphatases had a median of 4 NGIs per gene, relative to 2 NGIs for kinases. Thus, while yeast cells tolerate single deletion of phosphatases better than single deletion of kinases (Fig 1C), in a sensitized background yeast cells were as sensitive to phosphatase deletion as they were to kinase deletion, in agreement with the results of the *C*. *elegans* screen.

### Asymmetries are evident in other post-translational modification systems

Are the characteristics we observed above also present in other reversible post-translational modification systems? For this we turned to the histone acetylation system, which regulates transcription through the action of histone-acetyltransferases and histone-deacetylases, and the protein ubiquitination system, which regulates protein function and fate through the action of ubiquitin ligases and ubiquitin proteases. Henceforth, we refer to histone-acetyltransferases and ubiquitin ligases as 'writers' since they add the modification (similarly to kinases), and to histone-deacetylases and ubiquitin proteases as 'erasers' since they remove the modification (similarly to phosphatases). We focused on analyses of budding yeast where data were available. For other post translational modification systems we did not have enough data, or the number of genes was too small for a reliable comparison of the different properties.

**Fig 5:**
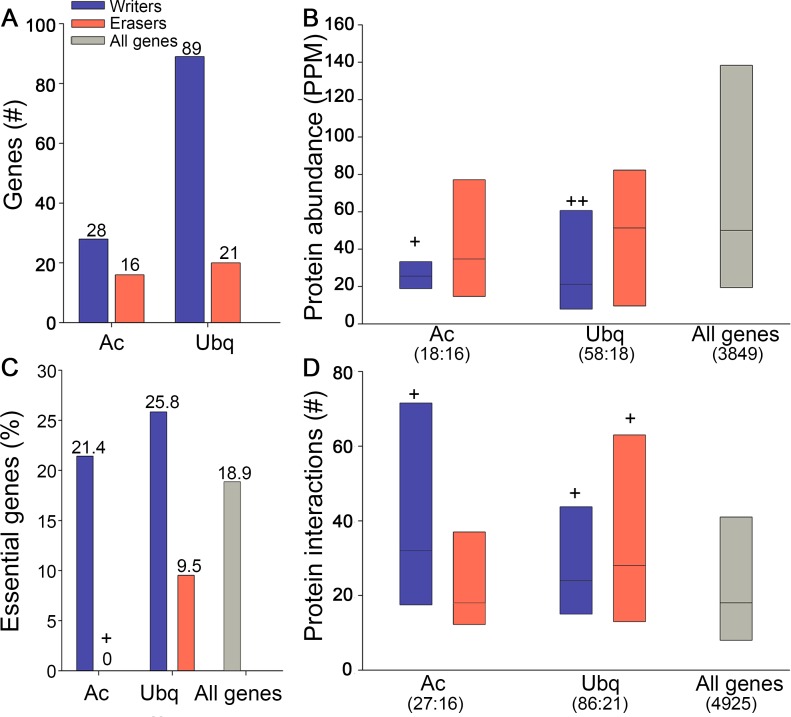
Writers and erasers of histone-acetylation (Ac) and protein-ubiquitination (Ubq) in budding yeast share features with kinases and phosphatases. A. Writer-coding genes are more abundant than eraser-coding genes. B. Writer proteins are significantly less abundant than all proteins (Ac p = 3.7*10^−3^, Ubq p = 1.4*10^−5^; Mann-Whitney test). C. Few eraser genes in budding yeast are essential (0% in acetylation, and 9.5% in the ubiquitination systems), in contrast to more than 21% of the writers (Ac p = 0.035; Fisher exact test). D. Acetylation writers, ubiquitination writers and ubiquitination erasers are significantly more connected by PPIs relative to all proteins (p = 0.0018, p = 0.0035 and p = 0.042, respectively, Mann-Whitney test). Box plots show the values at the first, second and third quartiles. ++ indicate p<10^−3^; */+ indicates p<0.05. In parenthesis are the numbers of writers and erasers for which data were available. Numbers above bars indicate Y-axis values.

Similarly to kinases and phosphatases, writers were encoded by many more genes relative to erasers (Fig 5A), and their protein products were significantly less abundant (Fig 5B). Furthermore, a much larger fraction of the writers were essential for survival (Fig 5C), and they had more PPI partners relative to all genes (Fig 5D). This suggests that these features reflect common design principles for these systems.

## Discussion

Historically, the study of protein phosphorylation concentrated mainly on the role of kinases at the expense of phosphatases [11]. Many kinases have been associated with specific functions and phenotypes, while phosphatases remained far less characterized. This difference has pharmaceutical implications that become apparent upon examining the targets of approved drugs: 17% of the human kinases were targeted by drugs, relative to only 6.5% of the human phosphatases [31]. However, it has been unclear whether this discrepancy is due to historical biases, or reflects a real difference in the organization and impact of kinases versus phosphatases. Here, we addressed this discrepancy systematically by analyzing data from unbiased 'omics' screens across genes, transcripts, proteins, interactions, and organisms.

Our first observation regards the quantitative relationships between kinases and phosphatases (Fig 1). Despite the seeming symmetry between kinases and phosphatases, which act on the same protein targets, eukaryotic genomes contain numerous kinase genes, over twice the number of phosphatase genes [4, 12]. Nonetheless, we show that a quantitative balance is maintained between kinases and phosphatases at the protein level, since the fewer phosphatase genes encode high-copy proteins. These high-copy proteins can be incorporated into different phosphatase complexes with distinct regulatory subunits, activities and specificities, as has been shown for some well-studied phosphatases [4, 11, 20].

Our second observation relates to the outcome of interference with kinases versus phosphatases. Given the numerous kinase genes per organism, one may hypothesize that interference with a specific kinase might lead to a kinase-specific response, whereas interference with a specific phosphatase might lead to a more general response. Indeed, this hypothesis is supported by our analyses of *C*. *elegans* vulva development (Fig 4). The phenotypes obtained upon knocking down individual kinase were variable and background-specific. In one background, some kinases had strong effects on vulva development, some had medium effects while other had no effect, whereas in another background, the effects of the same kinases were different. In contrast, knocking down any phosphatase gave a medium phenotype, with little variability among the phosphatases.

The scalable, variable impacts of kinases were not limited to a specific *C*. *elegans* system but also genome-wide, as we observed in budding yeast. Using hierarchical analysis, we identified a hierarchy of kinases that was not shown before (Fig 3). This hierarchy was relevant to the impact of kinases on the yeast phospho-proteome, transcriptome, and organism fitness. Notably, we did not observe a hierarchy of phosphatases. Together with the experimental analysis of *C*. *elegans*, this suggests that kinases have a broad range of specificities and impact that helps them mediate highly-specific responses to signals. This broad range makes kinases easier to characterize, and more favorable drug targets. In contrast, the relative uniformity among phosphatases implies that, in general, phosphatases are less favorable targets for controlling specific phenotypes. The discrepancy in the characterization of kinases at the expense of phosphatases is thus rooted in the different organization of each group.

Another feature that differed between kinases and phosphatases is their responsiveness (Fig 2). Kinases were more responsive and had a higher capacity to change at the gene and protein levels, making them specialized regulators that can rapidly alter their behavior and fine-tune cellular responses to signals. Phosphatases were less responsive, in agreement with the view that many phosphatases are catalytically active continuously [32]. We remind here that our analysis was focused on the catalytic subunits of kinases and phosphatases. The responsiveness and specificity of phosphatase complexes involves additional factors, including dynamic changes in their regulatory and inhibitory subunits, in the concentrations of relevant ions such as calcium, in their cellular localization, and more, as shown for the extensively studied PP1 and PP2A phosphatases [4, 11, 20]. The responsiveness of these factors and the elucidation of their effects on phosphatase activity are most intriguing, and their analysis will become feasible once more data are collected.

What are the benefits in having the catalytic subunits of phosphatases generally active, while kinases alter their activity in response to perturbations? One clear benefit is noise reduction: Kinases are less likely to trigger a response accidentally or prolong it, since the continuously active phosphatases can quickly attenuate the response. The serine/threonine phosphatase 1 (PP1) is a prominent example: this ubiquitous phosphatase acts as a "green" enzyme, promoting the recycling of proteins and the reversal of cells to basal and/or energy-conserving state [33]. This "driving with one foot on the break" strategy is common to many short-term processes, such as insulin signaling and glycogen metabolism, RAS GTPase signaling, activation of the transcription factor CREB and more [34].

**Fig 6:**
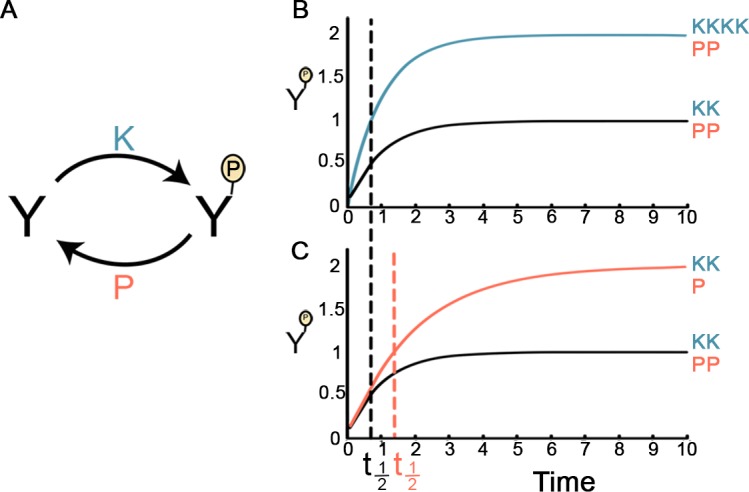
The dynamics of protein phosphorylation. A. Given a protein *Y*, the accumulation of its phosphorylated form, *Y*^*P*^, is determined by the activity rates of its kinase and phosphatase (*k* and *p*, respectively). B. The level of *Y*^*P*^ as a function of time (*t*). *Y*^*P*^ starts at zero and ends at its steady state level, described by the equation: *Y*^*P*^(*t*) = *k*/*p* * (1−*e*^−*pt*^). The black line depicts the regular activity rates (*k* = 1, *p* = 1, denoted KK and PP, respectively). The blue line depicts a 2-fold increase in the steady-state level of *Y*^*P*^, achieved by doubling the kinase activity (*k* = 2 and *p* = 1, denoted as KKKK and PP, respectively). The response time *t*_*1/2*_ (dashed black line) is identical in both cases. C. The black line is as described in B. The red line depicts a 2-fold increase in the steady-state level of *Y*^*P*^, achieved by 2-fold decrease in the phosphatase activity (*k* = 1, *p* = 0.5, denoted as KK and P, respectively). The response time *t*_*1/2*_ in this case (dashed red line) is twice the response time of the regular case.

Another feature that could be influenced by having phosphatases continuously active is phosphorylation dynamics. Below, we investigated phosphorylation dynamics as a function of the responsiveness of kinases and phosphatases. We analyzed a simplified scenario where a protein Y is reversibly phosphorylated. The concentration of the phosphorylated protein, denoted *Y*^*P*^, depends on the activity level of its kinase, denoted *k*, and the activity level of its phosphatase, denoted *p* (Fig 6A). Since the expression level of *Y* is often much higher than the level of its phosphorylated form *Y*^*P*^ [35–36], the change in the concentration of *Y* can be ignored, and the change in *Y*^*P*^ can be described by the following equation: *d Y*^*P*^*/ d*t = *k*–*p Y*^*P*^ ([37] and see Materials and Methods). The steady state level of *Y*^P^, denoted *Y*^*P*_*st*^, is thus given by: *Y*^*P*_*st*^ = *k* / *p*. Note that *Y*^*P*_*st*^ can be doubled in two ways: Either the kinase becomes twice more active (Fig 6B), or the phosphatase becomes only half active (Fig 6C). While both of these scenarios seem to lead to the same end result, the response time at which *Y*^*P*^ reaches *Y*^*P*_*st*^/2, denoted *t*_*1/2*_, is different [37]: The change in the *k* does not affect *t*_*1/2*_ (Fig 6B), whereas the change in *p* leads to a doubling of *t*_*1/2*_ (Fig 6C). Response time is a crucial factor in the ability of cells to adapt to changes in their environment. Thus, in this simplified scenario, keeping phosphatases intact, namely less responsive, and fine-tuning the activity of kinases, rendering them responsive, helps maintain timely responses to stimuli.

The asymmetry that we observed between kinases and phosphatases is inherent to their actions: although they act on the same targets, phosphatases always act after kinases do. We thus asked if other reversible cellular systems with writers and erasers behave similarly. We find that in budding yeast, the reversible systems of histone acetylation and protein ubiquitination show similar quantitative relationships and similar impact on fitness (Fig 5). Once suitable data become available, it will be intriguing to check whether hierarchical organization and differences in responsiveness are also maintained in those systems, whether other reversible systems such as protein methylation behave similarly, and what other features accompany this behavior.

In summary, we found that across eukaryotes kinases and phosphatases have an intriguing balance and are organized differently. Some of these features were previously demonstrated for specific kinases and phosphatases, and likewise, they may not describe every kinase and phosphatase. However, we show here quantitatively and broadly that these asymmetries hold widely in phosphorylation systems of diverse eukaryotes. They go hand-in-hand with the maintenance of transient, signal-specific responses, and provide insight into the different propensity of kinases and phosphatases to impact phenotypes.

## Materials and Methods

### Genes analyzed in this study

The annotations of genes to different molecular functions were obtained from the Gene Ontology (GO) Database [38]. We chose to work with GO annotations since they were in good agreement with other sources and provided a consistent framework across the different organisms. Kinases and phosphatases were defined as genes with molecular function annotation of 'protein kinase activity' (GO:0004672) or 'phosphoprotein phosphatase activity' (GO:0004721), respectively. Regulators of histone acetylation were defined as genes with 'histone acetyltransferase activity' (GO:0004402) or 'histone deacetylase activity' (GO:0004407) annotations. Regulators of protein ubiquitination were defined as genes with "ubiquitin-protein transferase activity" (GO:0004842) or "thiol-dependent ubiquitin-specific protease activity" (GO:0004843) annotations. For *H*. *sapiens* we considered only genes that were reviewed by UniProt database [39]. For *A*. *thaliana* we considered only genes with annotated TAIR accessions [40]. For *D*. *melanogaster* we considered only genes with annotated FlyBase accessions [41]. For *M*. *musculus* we considered only genes with annotated MGI accessions [42]. For *C*. *elegans* we considered all kinases and phosphatases annotated with multivulva or vulvaless phenotype according to WormBase [30]. To validate the trends we observed, we also analyzed curated kinases and phosphatases extracted from organism-specific databases, which showed similar results (S6 Fig).

### Data sources for protein expression, protein interactions and mutant phenotypes

Data of absolute protein expression levels were obtained through the PaxDb database. Data of *M*. *musculus* and of *H*. *sapiens* included the dataset designated 'integrated' in PaxDb [13]. Data of *S*. *cerevisiae* included the dataset of Ghaemmaghami et al [43]. Data of *D*. *melanogaster* included the dataset of Brunner et al [44] therein. Data of *A*. *thaliana* included the datasets of Baerenfaller et al. [45] and Castellana et al. [46] therein (in case multiple measurements were available for a protein the highest value was considered). Data of experimentally-verified physical associations between proteins were obtained from BIOGRID [47] (version 2.09) and INTACT [48]. For *A*. *thaliana* and *D*. *melanogaster*, we augmented these data with PPIs from TAIR [40, 49] and DroiD [50], respectively. Data of essential genes, whose inactivation or over-expression is deleterious to yeast, were obtained from SGD [51]. Data of essential genes in mouse were obtained from Mouse Genome Database (MGD) [42], similarly to Georgi et al [15]. Human genes associated with genetic diseases were identified through OMIM and were retrieved by their MIM Morbid Accession [16] using Ensembl BioMart [52].

### Yeast phosphorylation-related data sources

Data of known phosphorylation and de-phosphorylation events were obtained from Fielder et al [6], and consisted of high-confidence, manually-curated interactions from literature. Data of experimentally-determined phosphorylation sites per protein were obtained from Yachie et al [27]. Data of conserved phosphorylation sites were obtained from Minguez et al [19], limited to relative Residue Conservation Score (rRCS) > 95, as used therein for determining conservation. Data for the hierarchical analysis were obtained from Bodenmiller et al [9]. As defined therein, a kinase (or phosphatase) was considered to impact proteins that contained phospho-peptides that were differentially-phosphorylated in the kinase (phosphatase) inactivated strain. Data of differentially expressed genes in genetically-manipulated kinase (or phosphatase) strains were obtained from Kemmeren et al [17]. Data of protein half-life measures were obtained from Belle et al [18].

### Correlation between gene responsiveness and protein expression level in budding yeast

Gene responsiveness was calculated as the number of perturbations in which a gene was differentially expressed. We computed the Spearman correlation between this measure and protein expression level. There was no meaningful correlation upon considering all genes (r = -0.065), and upon considering only kinases and phosphatases (r = 0.11, p = 0.36).

### Hierarchy construction

Kinases were divided into layers according to the numbers of their impact-target relationships [9]. The top layer contained kinases that impacted kinases and were not targeted by any kinase. The middle layer contained kinases that impacted kinases and were also targeted by kinases. The bottom layer contained kinases that did not impact any kinase and were targeted by kinases. The outgroup contained kinases with no impact or target relationships with any kinase, and was further ignored. Phosphatases were incorporated into the hierarchy by considering impact relationships among phosphatases, and between phosphatases and kinases (S1 Table). The hierarchy in Fig 3A was depicted using Cytoscape 2.8 [53]. We tested the reproducibility of the features of each layer in two ways. Firstly, we repeated the analyses using the subset of fully-characterized kinases (S7 Fig), and observed similar trends (S4 Fig). Secondly, we recreated the hierarchy so that the assignment of a kinase to a layer will be more robust, i.e., will not depend on a single impact relationship. Specifically, a kinase was associated with (i) the top layer, if the kinase impacted two or more kinases, and was the target of at most one kinase (which could be noise); (ii) the middle layer, if it impacted two or more kinases and was targeted by two or more kinases; and (iii) the bottom layer, if it impacted at most one kinase and was targeted by two or more kinases. Again, the features of the original hierarchy were maintained (S5 Fig).

### Statistical analyses

The probability of overlap between datasets was calculated using Fisher's exact test (two tailed). The probability of observing similar distributions between two gene sets was calculated using Mann-Whitney test (one tailed). In all analyses, only measured genes/proteins were considered. Statistical tests were computed using Python 2.7, fisher 0.1.4 package and Scipy package. Statistical analysis of RNA interference experiments was performed using two-tailed Mann-Whitney test in STATISTICA. In all figures, star symbol (*) indicates a statistically significant difference between kinases and phosphatases, and plus symbol (+) indicates statistically significant difference between the respective group and the whole genome. The number of star/plus symbols indicates the significance level: ***/+++ indicates p<10^−6^; **/++ indicates p<10^−3^; */+ indicates p<0.05.

### Phosphorylation dynamics analysis

The change in the concentration of a phosphorylated protein *Y*^*P*^ results from two processes: the rate of accumulation of *Y*^*P*^ owing to the kinase activity rate, denoted *k*, and the rate of elimination of *Y*^*P*^ owing to the phosphatase activity rate, denoted *p*. The non-phosphorylated protein is often much more abundant than the phosphorylated protein *Y*^*P*^ [35–36], implying that the non-phosphorylated protein is generally not a rate-limiting factor. Therefore, the change in *Y*^*P*^ can be described by: *dY*^*P*^ */ d*t = *k*–*p Y*^*P*^ [37]. Following a signal that activates the kinase, *Y*^*P*^ accumulates and approaches its steady state as described by: *Y*^*P*^ (t) = Y^*P_st*^ *(1-e^-*p*t^) [37]. The response time *t*_*1/2*_ is derived from this equation upon solving it for *Y*^*P*^ (t) = Y^*P_st*^/2, resulting in *t*_*1/2* =_ log (2)/*p*.

### RNA interference (RNAi)

The experiment included RNAi for each kinase and phosphatase that was annotated with multivulva or vulvaless phenotype in WormBase [30]. Nematodes, *let-60(ga89)* and *bar-1(ga80)* (SD551 and EW15 strains, respectively), were grown on NGM plates seeded with the *Escherichia coli* OP50-1 strains at 15°C. In each experiment 15–30 embryos, laid at 15°C, were picked and transferred to fresh plates seeded with *E*. *coli* strain HT115(DE3) transformed with the indicated RNAi vectors (obtained from the Ahringer or Vidal RNAi libraries), as previously described [54]. SD551 animals were grown at the permissive temperature of 15°C until the first larval stage (L1) to avoid embryo lethality, and were then shifted to the partially restrictive temperature of 22.5°C until day 2 of adulthood. EW15 animals were grown in the partially restrictive temperature of 22.5°C until day 2 of adulthood. Animals showing Vul or Muv phenotypes were scored as defective vulva. Data are presented as percentage of animals showing defective vulva (Median ± SEM). mRNA levels were examined by quantitative RT-PCR to validate RNAi knock-down. RNAi experiments were repeated at least 3 times for the computation of SEM values. Total RNA was extracted from SD551 and EW15 animals fed with RNAi vectors using the TRIzol reagent (Invitrogene). For cDNA synthesis, mRNA was reverse-transcribed using the iScriptTM cDNA Synthesis Kit (Bio-Rad). Quantitative PCR was performed on a C1000 Thermal Cycler (Bio-Rad) with SsoFas EvaGreen Supermix (Bio-Rad). The primer sequences used in this procedure are given in S2 Table. RNAi knock-down of mRNA levels was controlled by comparing the mRNA levels of the target gene with mRNA levels of animals fed on bacteria containing the empty vector (pL4440).

## Supporting Information

S1 Fig Differences in gene numbers, protein abundance and essentiality between tyrosine-kinases and tyrosine-phosphatases. The list of tyrosine kinases and phosphatases was created by gathering from Gene Ontology (GO) proteins annotated to 'protein tyrosine kinase activity' (GO:0004713) and to 'protein tyrosine phosphatase activity' (GO:0004725). From these lists we excluded proteins that were also annotated to 'protein serine/threonine kinase activity' (GO:0004674) or 'protein serine/threonine phosphatase activity' (GO:0004722), thus leaving in only proteins that were exclusively tyrosine kinases and phosphatases.
ANumbers of genes coding for tyrosine-kinases and tyrosine-phosphatases in five eukaryotic genomes.BTyrosine-kinase proteins are significantly less abundant than all proteins in plant (p = 0.041; median abundance of tyrosine-kinases versus all proteins: 1.42: 3.8) and fly (p = 3.8*10^−5^; median abundance of tyrosine-kinases versus all proteins: 2.54: 12.97). They are also less abundant than tyrosine-phosphatases in human (p = 0.048; median abundance of tyrosine-kinases versus phosphatases 0.33: 0.62). In parenthesis are the numbers of kinases and phosphatases per organism for which data were available. Box plots show the values at the first, second and third quartiles. P-values were computed using Mann-Whitney test.CIn mouse, the percentage of tyrosine-phosphatases that are essential for survival is smaller than that of tyrosine kinases. In human, the percentage of tyrosine phosphatases that were associated with genetic diseases is significantly smaller than that of tyrosine-kinases (p = 0.006; Fisher exact test).
Yeast = *Saccharomyces cerevisiae*; Plant = *Arabidopsis thaliana*; Fly = *Drosophila melanogaster*; Mouse = *Mus musculus*; Human = *Homo sapiens*. ++/ indicates p<10^−3^; */+ indicates p<0.05.
(TIF)

S2 Fig The transcriptional responses and protein half-lives of kinases and phosphatases in budding yeast. AThe percentage of genes that are differentially expressed in at least one of the experiments reported by Kemmeren et al. [17]. The fraction of differentially expressed kinases and phosphatases is significantly lower relative to all genes (p = 0.0019, Fisher exact test).BKinase proteins have shorter half-lives relative to all proteins (p = 0.0026, Mann-Whitney test; median half-life in minutes: kinases = 33, phosphatases = 42, all genes = 44). Box plots show the values at the first, second and third quartiles. + indicates p<0.05.
(TIF)

S3 Fig Validations for the impact hierarchy of budding yeast kinases. AThe impact of kinases from each layer on the phospho-proteome. The numbers of proteins with altered phosphorylation upon kinase inactivation, for kinases from each layer, decreased upon moving down the kinase hierarchy. Inactivation of top-layer kinases affected the phosphorylation of significantly large sets of proteins (p = 0.0024), while inactivation of kinases from the bottom layer affected the phosphorylation of significantly small sets of proteins (p = 3.6*10^−5^). Inactivation of phosphatases affected the phosphorylation of significantly more proteins relative to the middle- and bottom-layer kinases (p = 0.0423). Statistical significance was computed using Mann-Whitney tests.BManually-curated kinase-kinase phosphorylations support the impact hierarchy. The percentages of kinases known to phosphorylate other kinases (outgoing) is significantly low in the bottom layer (p = 0.015), and the percentage of kinases known to be phosphorylated (incoming) is significantly low in the top layer (p = 0.041). Statistically significance was computed using Fisher exact test.CThe numbers of experimentally-verified phosphorylation sites harbored by kinases agrees with the impact hierarchy. Top-layer kinases harbor a significantly low number of phosphorylation sites relative to other layers (p = 2*10^−6^). Bottom-layer kinases harbor significantly more phosphorylation sites relative to top- and middle-layer kinases (p < 2.5*10^−4^). Statistical significance was computed using Mann-Whitney tests.DThe fraction of kinases harboring conserved phosphorylation sites is lowest in the top layer (p = 1.1*10^−4^) and highest in the bottom layer (p = 2.7*10^−3^). Statistical significance was computed using Fisher exact test.
TOP = top layer, MID = middle layer, BOT = bottom layer. ** indicates p<10^−3^; * indicates p<0.05. Each box-plot shows the values at the first, second and third quartiles. Statistical significance was computed of one layer relative to the two other layers.
(TIF)

S4 Fig The distinct impact of kinase layers when limiting each layer to kinases that were fully measured. Each layer contained only kinases that were both inactivated and detected in Bodenmiller et al. [9], including 14 top-layer kinases (TOP#), 26 middle-layer kinases (MID#), and 11 bottom-layer kinases (BOT#). Note that the middle layer was unchanged (MID# = MID), as all middle-layer kinases were fully measured.
AThe impact of kinases on phosphorylation of proteins, as measured by the numbers of proteins with altered phosphorylation upon kinase inactivation, for kinases from each layer, decreased upon moving down the kinase hierarchy. Bottom layer kinases affected significantly smaller sets of proteins relative to middle- and top-layer kinases (p = 1.3*10^−4^).BThe impact of kinases on gene expression, as measured by the numbers of differentially expressed genes upon kinase inactivation, for kinases from each layer, decreased upon moving down the kinase hierarchy. Bottom-layer kinases affected significantly smaller sets of genes relative to middle- and top-layer kinases (p = 0.028).CThe phenotypic impact of kinases, as measured by the percentage of essential kinase genes in each layer, is highest for top-layer kinases.
Statistical significance was calculated for one layer against the two other layers using the Mann-Whitney test. Box plots show the values at the first, second and third quartiles. ** indicates p<10^−3^; * indicates p<0.05.
(TIF)

S5 Fig The distinct impact of kinase layers upon reconstructing the hierarchy using more stringent thresholds. The revised hierarchy consisted of 216 kinase-kinase relationships involving 29 top-layer kinases (TOP*), 16 middle-layer kinases (MID*) and 24 bottom-layer kinases (BOT*).
AThe impact of kinases on phosphorylation of proteins, as measured by the numbers of proteins with altered phosphorylation upon kinase inactivation, for kinases from each layer, decreased upon moving down the kinase hierarchy. Top-layer kinases affect significantly larger sets of proteins (p = 9*10^−5^) and bottom layer kinases affect significantly smaller sets of proteins (p = 8*10^−6^).BThe impact of kinases on gene expression, as measured by the number of differentially expressed genes upon kinase inactivation, for kinases from each layer.CThe phenotypic impact of kinases, as measured by the percentage of essential kinase genes in each layer, is highest for top-layer kinases.
Statistical significance was calculated for each layer against the two other layers using the Mann-Whitney test. Box plots show the values at the first, second and third quartiles. ** indicates p<10^−3^.
(TIF)

S6 Fig Differences in gene numbers, protein abundance, essentiality and protein-protein interactions between curated kinases and phosphatases. We repeated our analyses using lists of curated kinases and phosphatases from the following organism-specific databases: Saccharomyces Genome Database (SGD) for yeast, The Arabidopsis Information Resource (TAIR) for plant, FlyBase for fly and Mouse Genome Informatics (MGI) for mouse. For human, we obtained data from PhosphoSitePlus website: Kinases were extracted as genes with 'kinase, protein' annotation, and phosphatases as genes with at least one of the following annotations: 'Protein phosphatase, dual-specificity', 'Protein phosphatase, Ser/Thr (non-receptor)', 'Protein phosphatase, tyrosine (non-receptor)' or 'Receptor protein phosphatase, tyrosine'.
AKinase-coding genes are more abundant than phosphatase-coding genes in the five eukaryotic genomes.BPhosphatase proteins are significantly more abundant than kinase proteins in the five eukaryotic proteomes. Median values for kinases, phosphatases and Mann-Whitney p-values per organism are as follows: yeast 30.4, 61.3, p = 0.008; plant 1.3, 4.1, p<10^−10^; fly 7.8, 10.6, p = 0.044; mouse 2.8, 6.3, p = 9*10^−4^; human 0.31, 0.4, p = 0.03. In parenthesis are the numbers of kinases and phosphatases per organism for which data were available.CThe fraction of phosphatases that are essential for survival (yeast and mouse), or were associated with genetic disease (human) is significantly smaller than that of kinases (yeast p = 0.043, mouse p = 0.0018, human p = 0.006; Fisher exact test).BKinases are significantly more involved in protein-protein interactions (PPIs) relative to phosphatases (*) or to all proteins (+). The numbers of kinases, phosphatases, and protein-coding genes for which PPI data were available, and the Mann-Whitney p-value per organism, are as follows: Yeast 127, 48, 4925, p<10^−10^; plant 382, 94, 6430, p = 0.006; fly 206, 82, 9539, p = 0.04; mouse 311, 52, 5527, p = 2*10^−9^; human 508, 135, 16,387, p = 3.2*10^−7^.
Box plots show the values at the first, second and third quartiles. Yeast = *Saccharomyces cerevisiae*; Plant = *Arabidopsis thaliana*; Fly = *Drosophila melanogaster*; Mouse = *Mus musculus*; Human = *Homo sapiens*. ***/+++ indicates p<10^−6^; ** indicates p<10^−3^; * indicates p<0.05.
(TIF)

S7 Fig The percentages of kinases and phosphatases that were characterized in the phospho-proteomic analysis of Bodenmiller et al.[9]. 'Detected' enzymes denote phosphorylation enzymes that contain a peptide whose abundance was measured. 'Inactivated' enzymes denote phosphorylation enzymes for which a strain carrying the inactivated enzyme was profiled. TOP = top layer, MID = middle layer, BOT = bottom layer, OUT = outgroup, PHO = phosphatases.
(TIF)

S1 Table A listing of yeast kinases and phosphatases and the association of kinases to layers in the impact hierarchy. (XLSX)

S2 Table Primer sequences used to measure mRNA levels for the validation of RNAi knock-down in *C*. *elegans*. (XLSX)
