# Supplementary figures and images for: An Asymmetrically Balanced Organization of Kinases versus Phosphatases across Eukaryotes Determines Their Distinct Impacts
Source: PLoS Comput Biol. 2017 Jan 30;13(1):e1005221. doi: 10.1371/journal.pcbi.1005221 (PMC5279721; doi:10.1371/journal.pcbi.1005221)

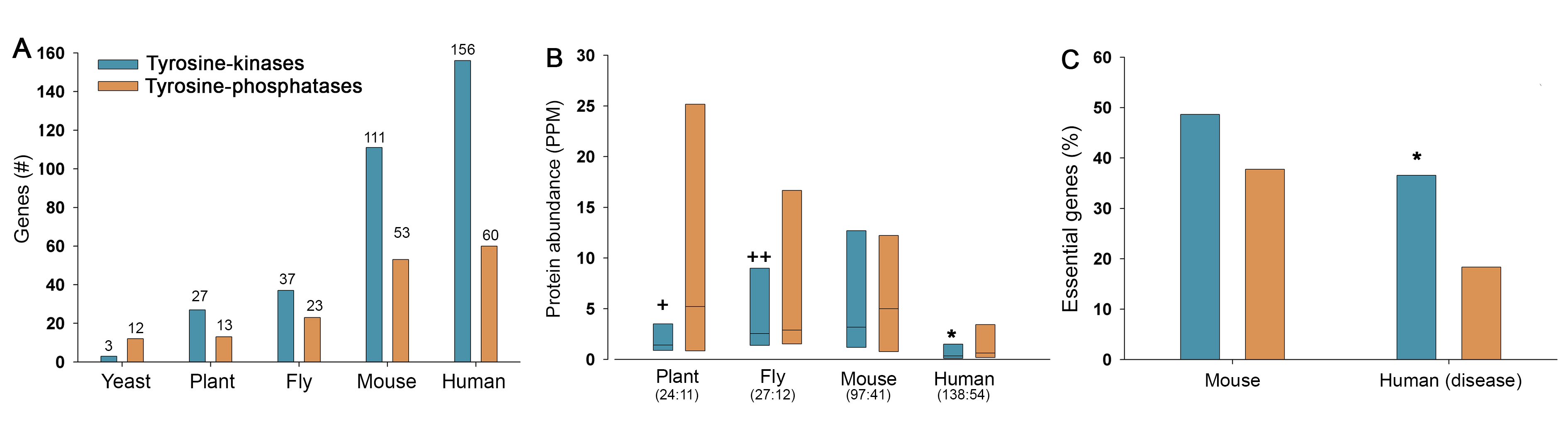

Supplement: S1 Fig — The list of tyrosine kinases and phosphatases was created by gathering from Gene Ontology (GO) proteins annotated to 'protein tyrosine kinase activity' (GO:0004713) and to 'protein tyrosine phosphatase activity' (GO:0004725). From these lists we excluded proteins that were also annotated to 'protein serine/threonine kinase activity' (GO:0004674) or 'protein serine/threonine phosphatase activity' (GO:0004722), thus leaving in only proteins that were exclusively tyrosine kinases and phosphatases. ANumbers of genes coding for tyrosine-kinases and tyrosine-phosphatases in five eukaryotic genomes.BTyrosine-kinase proteins are significantly less abundant than all proteins in plant (p = 0.041; median abundance of tyrosine-kinases versus all proteins: 1.42: 3.8) and fly (p = 3.8*10−5; median abundance of tyrosine-kinases versus all proteins: 2.54: 12.97). They are also less abundant than tyrosine-phosphatases in human (p = 0.048; median abundance of tyrosine-kinases versus phosphatases 0.33: 0.62). In parenthesis are the numbers of kinases and phosphatases per organism for which data were available. Box plots show the values at the first, second and third quartiles. P-values were computed using Mann-Whitney test.CIn mouse, the percentage of tyrosine-phosphatases that are essential for survival is smaller than that of tyrosine kinases. In human, the percentage of tyrosine phosphatases that were associated with genetic diseases is significantly smaller than that of tyrosine-kinases (p = 0.006; Fisher exact test). Yeast = Saccharomyces cerevisiae; Plant = Arabidopsis thaliana; Fly = Drosophila melanogaster; Mouse = Mus musculus; Human = Homo sapiens. ++/ indicates p<10−3; */+ indicates p<0.05. (TIF) [file pcbi.1005221.s001.tif]

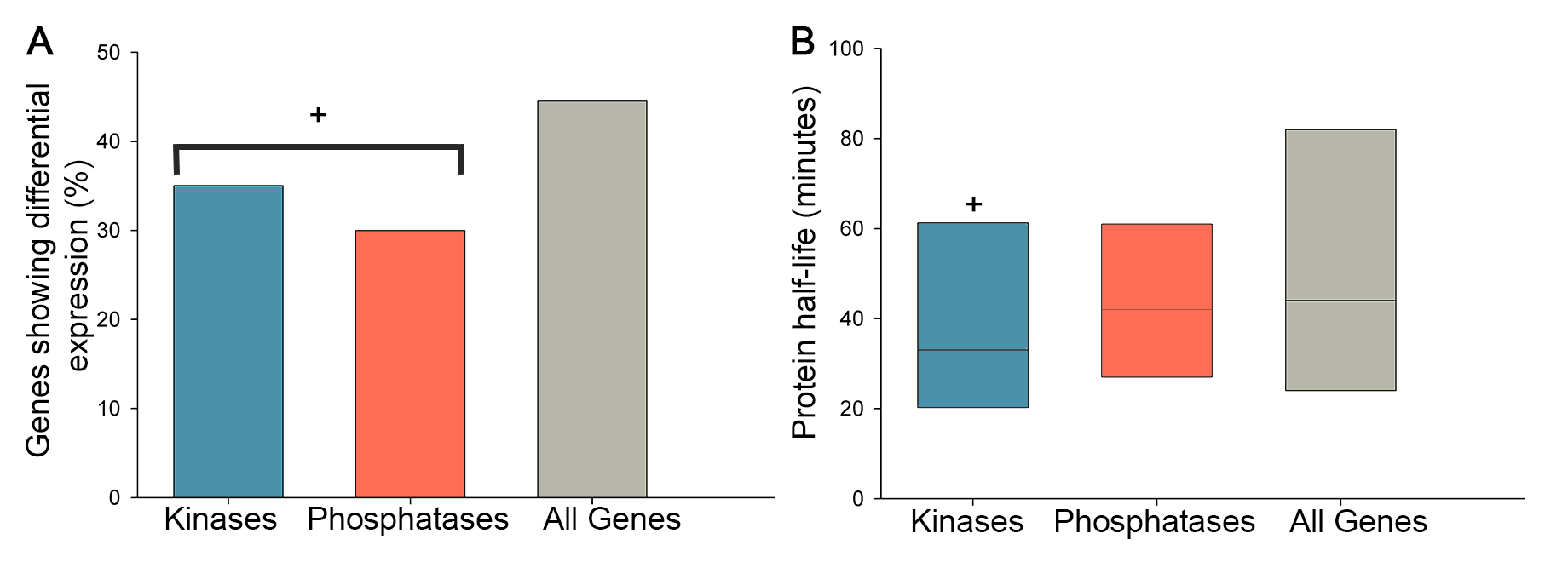

Supplement: S2 Fig — AThe percentage of genes that are differentially expressed in at least one of the experiments reported by Kemmeren et al. [17]. The fraction of differentially expressed kinases and phosphatases is significantly lower relative to all genes (p = 0.0019, Fisher exact test).BKinase proteins have shorter half-lives relative to all proteins (p = 0.0026, Mann-Whitney test; median half-life in minutes: kinases = 33, phosphatases = 42, all genes = 44). Box plots show the values at the first, second and third quartiles. + indicates p<0.05. (TIF) [file pcbi.1005221.s002.tif]

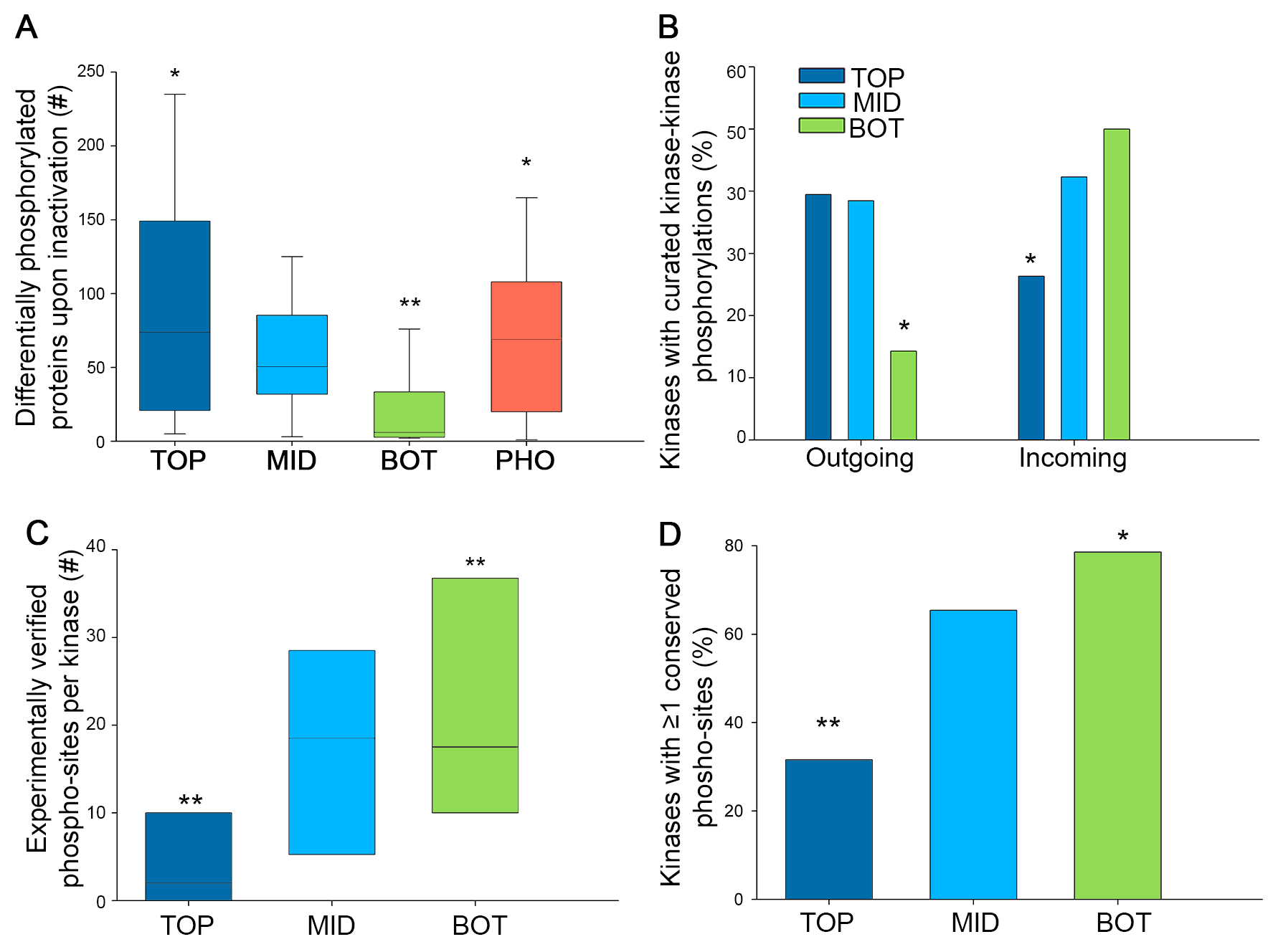

Supplement: S3 Fig — AThe impact of kinases from each layer on the phospho-proteome. The numbers of proteins with altered phosphorylation upon kinase inactivation, for kinases from each layer, decreased upon moving down the kinase hierarchy. Inactivation of top-layer kinases affected the phosphorylation of significantly large sets of proteins (p = 0.0024), while inactivation of kinases from the bottom layer affected the phosphorylation of significantly small sets of proteins (p = 3.6*10−5). Inactivation of phosphatases affected the phosphorylation of significantly more proteins relative to the middle- and bottom-layer kinases (p = 0.0423). Statistical significance was computed using Mann-Whitney tests.BManually-curated kinase-kinase phosphorylations support the impact hierarchy. The percentages of kinases known to phosphorylate other kinases (outgoing) is significantly low in the bottom layer (p = 0.015), and the percentage of kinases known to be phosphorylated (incoming) is significantly low in the top layer (p = 0.041). Statistically significance was computed using Fisher exact test.CThe numbers of experimentally-verified phosphorylation sites harbored by kinases agrees with the impact hierarchy. Top-layer kinases harbor a significantly low number of phosphorylation sites relative to other layers (p = 2*10−6). Bottom-layer kinases harbor significantly more phosphorylation sites relative to top- and middle-layer kinases (p < 2.5*10−4). Statistical significance was computed using Mann-Whitney tests.DThe fraction of kinases harboring conserved phosphorylation sites is lowest in the top layer (p = 1.1*10−4) and highest in the bottom layer (p = 2.7*10−3). Statistical significance was computed using Fisher exact test. TOP = top layer, MID = middle layer, BOT = bottom layer. ** indicates p<10−3; * indicates p<0.05. Each box-plot shows the values at the first, second and third quartiles. Statistical significance was computed of one layer relative to the two other layers. (TIF) [file pcbi.1005221.s003.tif]

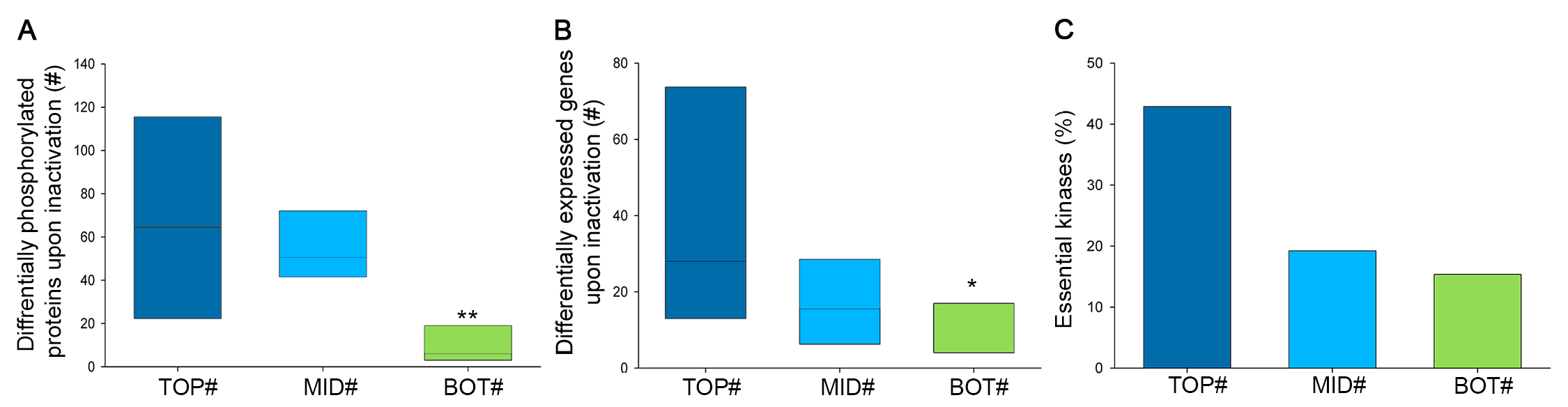

Supplement: S4 Fig — Each layer contained only kinases that were both inactivated and detected in Bodenmiller et al. [9], including 14 top-layer kinases (TOP#), 26 middle-layer kinases (MID#), and 11 bottom-layer kinases (BOT#). Note that the middle layer was unchanged (MID# = MID), as all middle-layer kinases were fully measured. AThe impact of kinases on phosphorylation of proteins, as measured by the numbers of proteins with altered phosphorylation upon kinase inactivation, for kinases from each layer, decreased upon moving down the kinase hierarchy. Bottom layer kinases affected significantly smaller sets of proteins relative to middle- and top-layer kinases (p = 1.3*10−4).BThe impact of kinases on gene expression, as measured by the numbers of differentially expressed genes upon kinase inactivation, for kinases from each layer, decreased upon moving down the kinase hierarchy. Bottom-layer kinases affected significantly smaller sets of genes relative to middle- and top-layer kinases (p = 0.028).CThe phenotypic impact of kinases, as measured by the percentage of essential kinase genes in each layer, is highest for top-layer kinases. Statistical significance was calculated for one layer against the two other layers using the Mann-Whitney test. Box plots show the values at the first, second and third quartiles. ** indicates p<10−3; * indicates p<0.05. (TIF) [file pcbi.1005221.s004.tif]

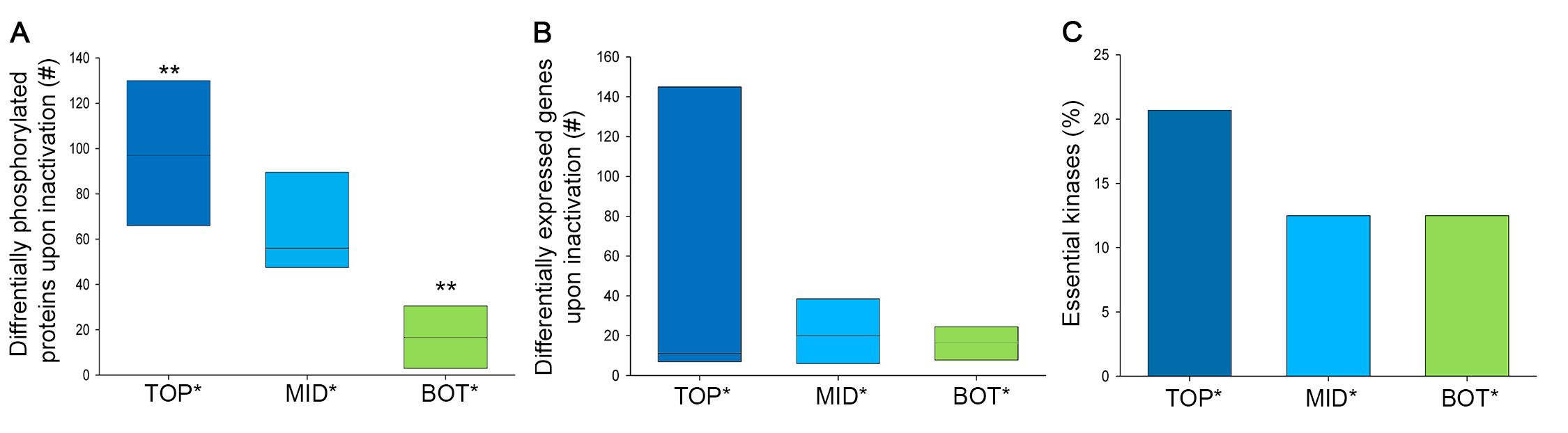

Supplement: S5 Fig — The revised hierarchy consisted of 216 kinase-kinase relationships involving 29 top-layer kinases (TOP*), 16 middle-layer kinases (MID*) and 24 bottom-layer kinases (BOT*). AThe impact of kinases on phosphorylation of proteins, as measured by the numbers of proteins with altered phosphorylation upon kinase inactivation, for kinases from each layer, decreased upon moving down the kinase hierarchy. Top-layer kinases affect significantly larger sets of proteins (p = 9*10−5) and bottom layer kinases affect significantly smaller sets of proteins (p = 8*10−6).BThe impact of kinases on gene expression, as measured by the number of differentially expressed genes upon kinase inactivation, for kinases from each layer.CThe phenotypic impact of kinases, as measured by the percentage of essential kinase genes in each layer, is highest for top-layer kinases. Statistical significance was calculated for each layer against the two other layers using the Mann-Whitney test. Box plots show the values at the first, second and third quartiles. ** indicates p<10−3. (TIF) [file pcbi.1005221.s005.tif]

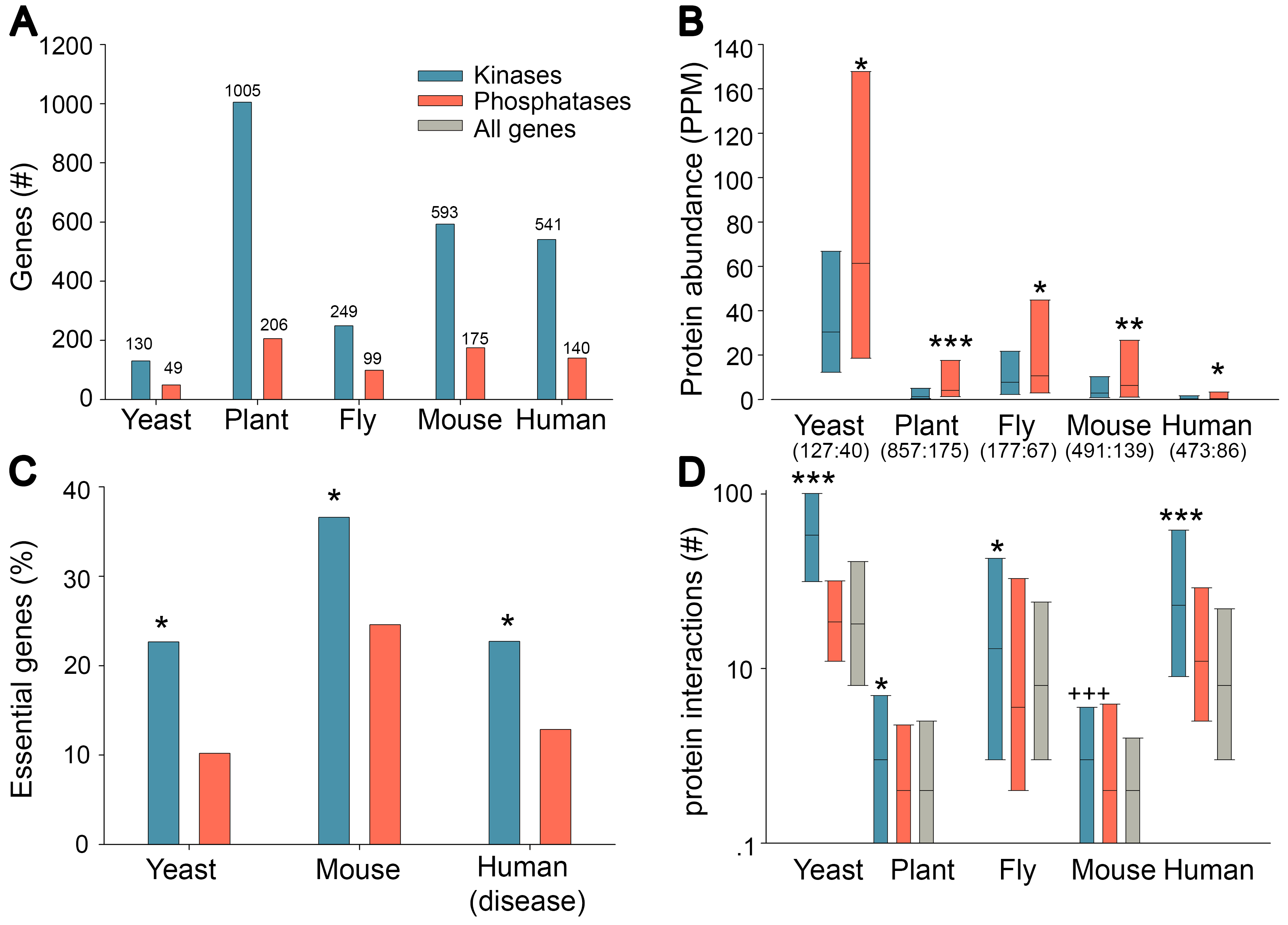

Supplement: S6 Fig — We repeated our analyses using lists of curated kinases and phosphatases from the following organism-specific databases: Saccharomyces Genome Database (SGD) for yeast, The Arabidopsis Information Resource (TAIR) for plant, FlyBase for fly and Mouse Genome Informatics (MGI) for mouse. For human, we obtained data from PhosphoSitePlus website: Kinases were extracted as genes with 'kinase, protein' annotation, and phosphatases as genes with at least one of the following annotations: 'Protein phosphatase, dual-specificity', 'Protein phosphatase, Ser/Thr (non-receptor)', 'Protein phosphatase, tyrosine (non-receptor)' or 'Receptor protein phosphatase, tyrosine'. AKinase-coding genes are more abundant than phosphatase-coding genes in the five eukaryotic genomes.BPhosphatase proteins are significantly more abundant than kinase proteins in the five eukaryotic proteomes. Median values for kinases, phosphatases and Mann-Whitney p-values per organism are as follows: yeast 30.4, 61.3, p = 0.008; plant 1.3, 4.1, p<10−10; fly 7.8, 10.6, p = 0.044; mouse 2.8, 6.3, p = 9*10−4; human 0.31, 0.4, p = 0.03. In parenthesis are the numbers of kinases and phosphatases per organism for which data were available.CThe fraction of phosphatases that are essential for survival (yeast and mouse), or were associated with genetic disease (human) is significantly smaller than that of kinases (yeast p = 0.043, mouse p = 0.0018, human p = 0.006; Fisher exact test).BKinases are significantly more involved in protein-protein interactions (PPIs) relative to phosphatases (*) or to all proteins (+). The numbers of kinases, phosphatases, and protein-coding genes for which PPI data were available, and the Mann-Whitney p-value per organism, are as follows: Yeast 127, 48, 4925, p<10−10; plant 382, 94, 6430, p = 0.006; fly 206, 82, 9539, p = 0.04; mouse 311, 52, 5527, p = 2*10−9; human 508, 135, 16,387, p = 3.2*10−7. Box plots show the values at the first, second and third quartiles. Yeast = Saccharomyces cerevi [file pcbi.1005221.s006.tif]

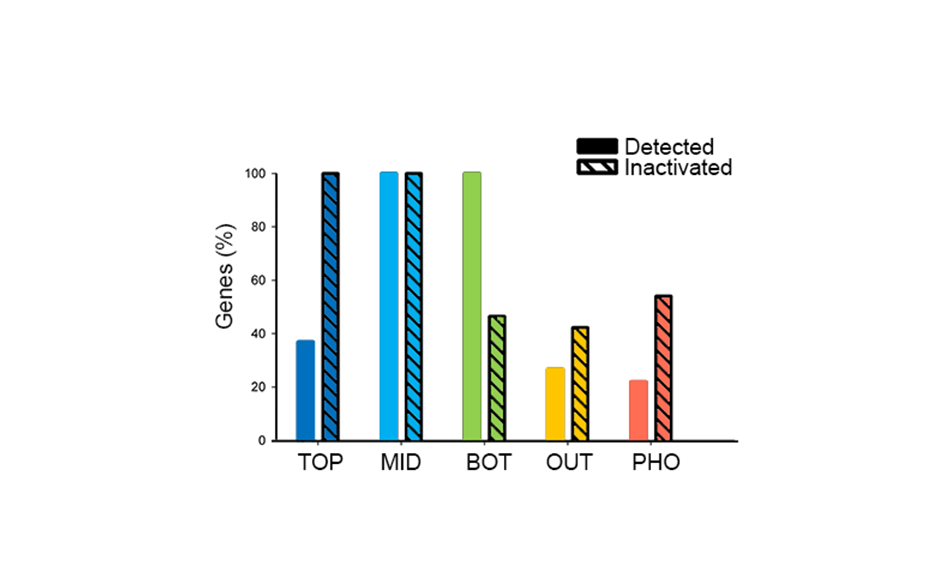

Supplement: S7 Fig — 'Detected' enzymes denote phosphorylation enzymes that contain a peptide whose abundance was measured. 'Inactivated' enzymes denote phosphorylation enzymes for which a strain carrying the inactivated enzyme was profiled. TOP = top layer, MID = middle layer, BOT = bottom layer, OUT = outgroup, PHO = phosphatases. (TIF) [file pcbi.1005221.s007.tif]
